# Assessing the Impact of Urbanization on Direct Runoff Using Improved Composite CN Method in a Large Urban Area

**DOI:** 10.3390/ijerph15040775

**Published:** 2018-04-17

**Authors:** Chunlin Li, Miao Liu, Yuanman Hu, Tuo Shi, Min Zong, M. Todd Walter

**Affiliations:** 1 CAS Key Laboratory of Forest Ecology and Management, Institute of Applied Ecology, Chinese Academy of Sciences, Shenyang 110016, China; lichunlin@iae.ac.cn (C.L.); huym@iae.ac.cn (Y.H.); tuoshi0411@163.com (T.S.); zongminzm@163.com (M.Z.); 2 University of Chinese Academy of Sciences, Beijing 100049, China; 3 Department for Biological and Environmental Engineering, Cornell University, 62 Riley-Robb Hall, Ithaca, NY 14853, USA; mtw5@cornell.edu

**Keywords:** direct runoff, soil conservation service, improved composite curve number, hydrologic impact, urbanization

## Abstract

Urbanization is one of the most widespread anthropogenic activities, which brings a range of physical and biochemical changes to hydrological system and processes. Increasing direct runoff caused by land use change has become a major challenge for urban ecological security. Reliable prediction of the quantity and rate of surface runoff is an inherently difficult and time-consuming task for large ungauged urban areas. In this study, we combined Geographic Information System and remote sensing technology with an improved Soil Conservation Service curve number model to evaluate the effects of land use change on direct runoff volume of the four-ring area in Shenyang, China, and analyzed trends of direct runoff at different scales. Through analyzing trends of direct runoff from 1984 to 2015 at different scales, we explored how urbanization and other potential factors affect direct runoff changes. Total direct runoff volume increased over time, and trends varied from the inner urban area to suburban area. Zones 1 and 2 had a tendency toward decreasing direct runoff volume and risks, while Zones 3 and 4 showed gradual increases at both regional and pixel scales. The most important influence on direct runoff change was urban surface change caused by urbanization. This study presents a framework for identifying hotspots of runoff increase, which can provide important guidance to urban managers in future green infrastructure planning, in the hopes of improving the security of urban water ecological patterns.

## 1. Introduction

Growing populations and economies are driving expansion of urban built-up areas in the form of urbanization across the globe and, by 2050, over 70% of the world’s population is expected to live in urban areas [1]. As urbanization progresses, urban land use changes, augmenting the area of impervious surfaces and, as a consequence, reducing infiltration during storm events and increasing direct runoff that eventually alters urban hydrologic processes [2,3,4]. Large volumes of direct runoff generally increase the frequency and grade of flooding, which increases potential property damage [2,5]. Surface direct runoff is an important parameter used in hydraulic engineering projects associated with flood protection [6]. Because rainfall–runoff is a complex and dynamic process, direct runoff is generally affected by precipitation, evapotranspiration, substrate permeability, soil moisture, and other factors [7,8,9]. Under similar rainfall conditions, more than 40% of rainfall can be converted to direct surface runoff in urban landscapes with more than 50% impervious surfaces. However, runoff in woodland areas may be as low as 13% [10,11]. Increasing direct runoff caused by urbanization is a major challenge for urban ecological security that calls for integrated storm water management solutions in urban planning and development [12].

The estimation of stormwater quantity has been a requirement in evaluating compliance of stormwater management regulations and in implementing effective control measures [13]. Many studies have used field measurements, experimental watersheds, and statistical modeling to evaluate the impact of urbanization on surface direct runoff [1,14,15,16]. However, reliable prediction of the quantity and rate of surface runoff is an inherently difficult and time-consuming task for large ungauged watersheds [17]. Under these circumstances, hydrologic modeling is an effective solution that can use limited data to simulate runoff volume [18,19,20]. Among all hydrologic models, the Soil Conservation Service curve number (SCS-CN, U.S. Department of Agriculture, Bronx, NY, USA) method is one of the most widely used empirical hydrologic models for computing the volume of direct surface runoff.

SCS-CN (renamed Natural Resources Conservation Services curve number; United States Department of Agriculture 1994) was developed by the U.S. Department of Agriculture in 1954 [21]. It can predict direct runoff from an expression for a rainfall–runoff curve that varies according to a single parameter called the curve number (CN). The dimensionless CN parameter describes the antecedent potential water retention of a watershed [22]. The method has become widely used because the CN value is tabulated for a variety of hydrologic conditions, land use types, and soil types. Consequently, these CN tables make it easy to transfer GIS (Geographic Information Systems, ESRI, Redlands, CA, USA) data into a rainfall–runoff model based on the SCS-CN method [8]. Ponce and Hawkins (1996) highlighted the advantages of the SCS model in terms of its simplicity, stability, good predictability and accuracy to reflect the watershed characteristic for runoff depth prediction [23]. The SCS-CN method since its inception has been widely accepted by scientist, hydrologists, water resources planners, agriculturists, foresters, and engineers for estimation of surface runoff [24]. Many distributed hydrologic models (such as SWAT (Soil and Water Assessment Tool), SWMM (Storm Water Management Model), HEC-HMS (Hydrologic Modeling System)) use the SCS-CN model as the hydrologic module to estimate the rainfall–runoff process.

Although many researchers have studied the effect of land use change on hydrologic cycles, most previous studies have focused on the watershed [25,26,27,28,29,30,31] or small urban catchment [32,33,34,35] scales. There have been few investigations into the direct runoff of a large urban area with high spatial resolution. This is because urban land use classification is very complicated and different land use types have different hydrologic characteristics. However, urban land cover except water can be generalized into three components (vegetation, impervious and soil) combined with a certain proportion according to the vegetation-impervious surface-soil (V-I-S) model presented by Ridd in 1995 [36]. V-I-S can greatly simplify complex urban land use types. Combined with V-I-S and remote sensing inversion, we can obtain the proportion of urban land use types in various periods using remote sensing images. The composite CN value of each pixel is calculated by an improved composite CN method according to the classification and proportion of the three components. Then, direct runoff in different periods can be calculated for a large urban area by the composite CN value and SCS-CN model.

As an established industrial base in northeastern China, Shenyang is experiencing rapid urbanization. The area of impervious surfaces is increasing rapidly because of various development policies in recent years, including the reform and opening-up policy and revitalization of the old northeastern industrial base policy. The process of urbanization affects the changes in land use and thus further affects the direct runoff. Therefore, cities in different urbanization stages will have different trends in runoff changes. As a result, increasing direct surface runoff has become a serious problem in the Shenyang urban area. This study used Shenyang as a case study to explore the impact of urbanization on direct runoff. In doing so, this study had several goals: (1) to develop an effective and efficient method to simulate direct runoff in the Shenyang urban area; (2) to explore trends of direct runoff from 1984 to 2015 at different scales; and (3) to analyze potential factors behind direct runoff changes.

## 2. Materials and Methods

### 2.1. Study Area and Data

**Figure 1:**
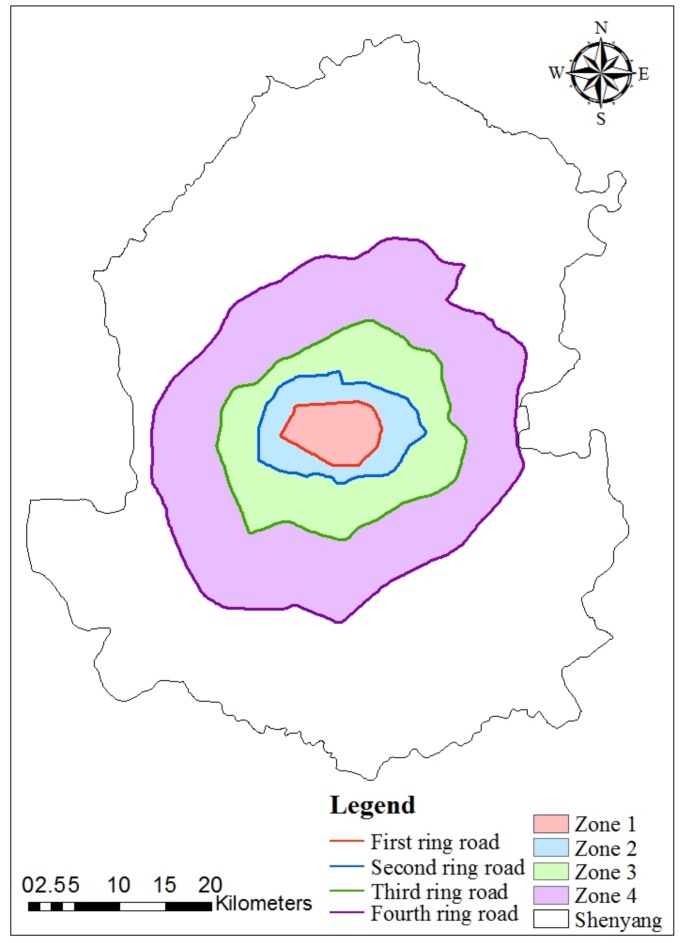
Regional location of study area, with four zones divided by four ring roads. Zone 1 is encircled by the first ring road. Zone 2 is the area between the first and second ring roads. Zone 3 represents the area between the second and third ring road. Zone 4 is the area between the third and fourth ring road. The four-ring area is the entire area of Zones 1–4.

Shenyang is the largest and most important industrial city in Northeast China (41°11′51″–43°02′13″N, 122°25′09″–123°48′24″E). The city has a temperate continental monsoon climate, with an average temperature of 8.1 °C and distinct seasons. Mean annual precipitation is 510–680 mm, most of which falls from June to August. The prevailing wind direction in summer is southwest. The urban sprawl of Shenyang is principally gradually expanding from the central to suburban areas, and the four-ring road network was created during this period. The entire urban area of Shenyang (called the four-ring area) can be divided into four areas (Zones 1–4) according to the four ring roads (Figure 1). From the first to fourth ring areas, the process of urbanization is represented. Our study area is the four-ring area, and water area was excluded because it cannot provide direct runoff. This area is the fastest growing urbanization part of Shenyang. Most of the population and built-up areas are within this area.

The annual changes of urban land use are not significant. Therefore, this study evaluated the land use every five years to calculate the changes of runoff. Considering the quality and availability of satellite remote sensing images, we chose Landsat 5 TM (Thematic Mapper) images for the years 1984, 1989, 1995, 2000, 2006 and 2010, and a Landsat 8 OLI (Operational Land Imager) image for the year 2015 to estimate proportions of impervious surface, vegetation, and soil through remote sensing inversion. Landsat images were obtained from the United States Geological Survey with acquisition dates from July to September, when it is easier to distinguish vegetation information. Because our study focused on the influence of land use change on runoff generation and the study area is 1200 km^2^, we do not consider the spatial heterogeneity of single rainfall. So, it was assumed that rainfall is homogeneous across the study area. Daily rainfall data of 2015 were obtained from a meteorological station in Shenyang. Total rainfall in 2015 was 573.2 mm, and the rain amount from May to October 2015 was 436.7 mm, or 76.2% of the annual total. The winter precipitation in Shenyang is mainly snowfall, and most precipitation in Shenyang is from May to October. The purpose of the present study was to explore the impact of urbanization on direct runoff, so we used the same rainfall data to simulate different periods of runoff. Therefore, daily rainfall data from May to October 2015 were used to simulate direct runoff in 1984, 1989, 1995, 2000, 2006, 2010, and 2015.

### 2.2. Direct Runoff Simulation Method

The modified SCS-CN model was used to simulate direct runoff from the urban area. Antecedent moisture condition (AMC) of soil is an index of watershed wetness [37]. There are three levels of AMC in practice, which could be physically unreasonable for sudden jumps in curve numbers, producing corresponding jumps in estimated runoff [38]. The Mishra and Singh (MS) model advantageously uses a separate expression based on antecedent 5-day rainfall to estimate antecedent moisture. This obviates sudden jumps in CN variation and, in turn, variation in retention capacity [39]. The MS model equations are as follows.

(1) $$Q=\{\begin{array}{c} \frac{\left(P-I_{a}\right)\left(P-I_{a}+M\right)}{P-I_{a}+M+S},P\geq I_{a}\\ 0,P<I_{a} \end{array},$$

(2) $$M=\{\begin{array}{c} \frac{S_{I}\left(P_{5}-0.2S_{I}\right)}{P_{5}+\left(1-0.2\right)S_{I}},P_{5}\geq 0.2S_{I}\\ 0,P_{5}<0.2S_{I} \end{array},$$

where *Q* is direct runoff depth (mm); *P* is precipitation depth (mm); *S* is potential maximum soil moisture retention (mm); *I_a_* is the initial abstraction (mm), which is often set to 0.2*S*; *M* is antecedent moisture (mm); *P*_5_ is antecedent 5-day rainfall (mm); and *S_I_* is potential maximum retention in dry condition (AMC I). *S* is defined by the dimensionless CN parameter. *S_I_* can be taken as approximately equal to the absolute potential maximum retention (*S*_0_):

(3) $$S_{I}=S_{0}=S+M,$$

(4) $$S=\frac{25400}{CN}-254.$$

We simulated direct runoff with the MS model. Actual daily rainfall data of 2015 were input to the model. ArcGIS was used to simulate the direct runoff of land use change caused by urbanization from 1984 to 2015 of Shenyang.

### 2.3. Linear Spectral Mixture Analysis

Linear spectral mixture analysis (LSMA) has been widely used in surface estimation, which implies that land cover is a combination of different surface materials [40,41]. LSMA is a physically deterministic modeling approach that decomposes the signal measured at a given pixel into its component parts called endmembers. It assumes that the reflectance of a single pixel in each spectral band is a linear combination of the characteristic reflectance of each endmember and their respective abundances [42,43]. We used the LSMA method to generalize urban land use types into three basic elements based on the V-I-S model, vegetation, impervious surface, and bare soil. The proportion of impervious surface, vegetation and soil of each pixel can be used to calculate the CN values.

To verify the accuracy of the proportions of vegetation, impervious surface and soil components, we selected 50 random points with area 300 m × 300 m. For each sample point, the vegetation, impervious surface and soil of 2015 and 2010 were visually interpreted on high-resolution images from Google Earth. Accuracy of the vegetation, impervious surface and soil maps were assessed by comparing the visual interpretation proportions from Google Earth and LSMA results. Root mean square error (RMSE) was computed to evaluate the accuracy of the un-mixing results. RMSE is a commonly used method for evaluating the difference between simulated and measured values. RMSE can be expressed by:

(5) $$RMSE=\sqrt{\frac{\sum_{i=1}^{N}\left(X_{i}-Y_{i}\right)}{N}},$$

where *X_i_* represents the estimated impervious surface, vegetation, and soil fractions of sample *i* from Landsat by LSMA; *Y_i_* is the digitized proportion of *i* from the high-resolution image; and *N* is the number of samples.

### 2.4. Improved Composite CN Method

An improved composite CN method proposed by Fan (2013) was used to calculate composite CN [17]. Each 30 m × 30 m pixel was assumed to be an independent drainage area and comprised impervious surface, vegetation and soil only. The composite CN value of each pixel can be computed as the area-weighted average of the CN values of impervious surface, vegetation and soil. The composite CN calculation is via:

(6) $$CN_{C}=S_{I}\times CN_{I}+S_{V}\times CN_{V}+S_{S}\times CN_{S},$$

where *CN_C_* is the composite CN value; *S_I_*, *S_V_*, and *S_S_* are fractions of impervious surface, vegetation and soil extracted by the LSMA, respectively; and *CN_I_*, *CN_V_*, and *CN_S_* are the initial CN values of impervious surface, vegetation and soil, respectively.

The composite CN was calculated under the dry antecedent moisture condition (AMC-I). *CN_I_* under AMC-I was assigned a value of 98 according to the lookup table of Technical Release 55 (TR-55). Before vegetation classification, we randomly selected 100 sites on Google Maps and manually divided them into four vegetation categories (good condition, fair condition, poor condition, and non-vegetated), and then calculated the NDVI (Normalized Difference Vegetation Index) values for each site. Through calculation, we got the division value of NDVI for vegetation classification. The vegetation was classified into four categories according to values of NDVI, with larger than 0.7 representing vegetated area in good condition, 0.4–0.7 representing fair conditions, 0–0.4 representing poor conditions, and less than 0 representing a non-vegetated area. Sand, silt, and clay are the three basic components, and the proportions of each component directly affect the infiltration of soil. Thus, the soil was classified into four hydrologic soil groups (A, B, C, and D) based on the proportion of sand and clay derived from the soil texture map of Shenyang. Regarding TR-55, initial values of *CN_V_* and *CN_S_* in AMC-I are shown in Table 1 and Table 2.

Slope is an important effect on CN. The higher the slope value is, the faster CN increases. Large CN values with large slope values may produce more surface runoff and little infiltration under the same conditions [44]. The underlying surface of the entire study area is relatively flat; only 3.6% of the area has a slope greater than 5%. Therefore, the influence of slope on CN was not considered.

### 2.5. Trend and Risk Analysis for Direct Runoff

Trends at regional scale were analyzed by calculating the annual runoff volume of four zones. Trends at pixel scale were produced using the ordinary least-squares regression method for direct runoff of each pixel to quantify the magnitude of the trends, using

(7) q=a+bt,

(8) $$b=\frac{\sum_{i=1}^{7}\left(t_{i}-\bar{t})(q_{i}-\bar{q}\right)}{\sum_{i=1}^{7}{\left(t_{i}-\bar{t}\right)}^{2}},$$

where *q* is direct runoff (mm); *t* is the year; $\bar{q}$ and $\bar{t}$ are mean values of *q* and *t*, respectively; *b* represents the trend magnitude; and *a* is the intercept.

The ordinary least-squares regression method is widely used in analyzing trends. Positive and negative *b* values represent increase and decrease in runoff, while the absolute value of *b* represents the rate of change. To investigate the trends of direct runoff, trends from 1984 to 2015 on a per-pixel basis were examined based on the equations. Direct runoff from 1984 to 2015 had an increasing trend when *b* > 0 and a decreasing trend when *b* < 0. The larger the absolute value of *b*, the faster the change of direct runoff. To further detect the extent of spatial trends, the *b*-value was divided into four levels according to its range: fast decrease (*b* < −5), slow decrease (−5 ≤ *b* ≤ 0), slow increase (0 < *b* ≤ 5), and fast increase (*b* > 5). Significance level is an important index to indicate the significance of spatial trends of direct runoff. We used the 5% significance level to determine the significance level of runoff trends over time: significant (*p* < 0.05) and non-significant (*p* ≥ 0.05). Direct runoff risk was divided into four degrees according to the annual mean runoff coefficient (r_c_): extremely high (r_c_ > 0.5), high (0.4 < r_c_ ≤ 0.5), medium (0.3 < r_c_ ≤ 0.4) and low (r_c_ ≤ 0.3).

## 3. Results and Discussion

### 3.1. LSMA Accuracy Assessment

Urban surfaces are very complex and runoff coefficients for different surface types are very different. Thus, simplifying the urban surface as three basic types make calculation easy, but this increases uncertainty in the predicted direct runoff. Assessment results indicate good agreement between estimation and interpretation. RMSEs for vegetation, impervious surface and soil were 0.19, 0.21, and 0.24, respectively. The accuracy estimations of vegetation, impervious surface and soil are good enough for calculating direct runoff. However, the difference in quality of remote sensing images acquired by different satellite remote sensors may cause some uncertainty in the final direct runoff estimation. The amount of cloud cover in those images and soil moisture conditions may also impact estimates of land use types by LSMA.

### 3.2. Direct Runoff Volume and Risk

**Figure 2:**
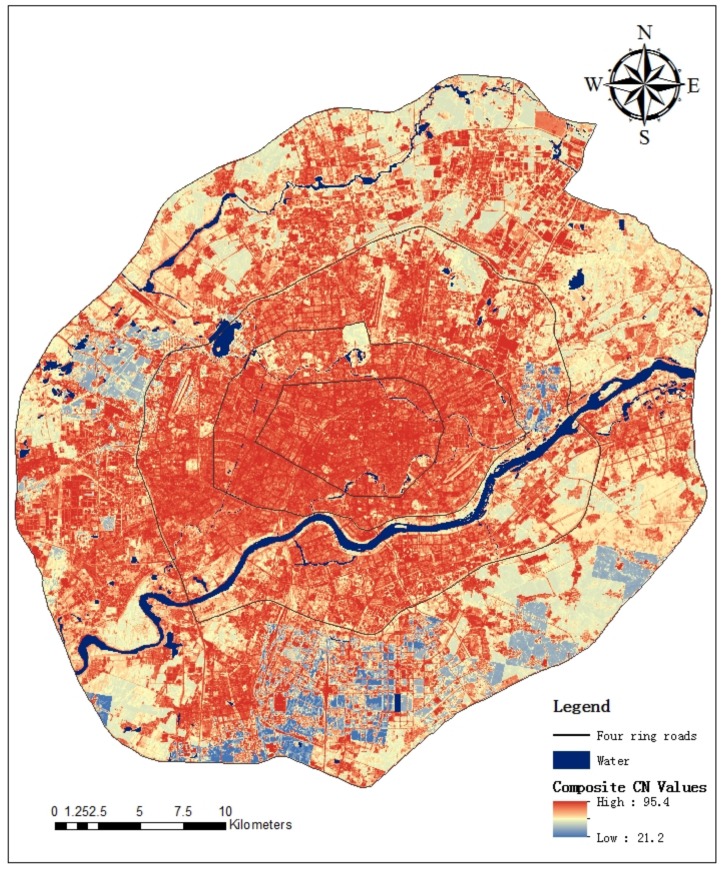
Composite CN values of Shenyang urban area in 2015 under AMC-I condition.

Composite CN values of Shenyang urban area in 2015 under AMC-I condition were shown in Figure 2. Areas with high CN values were concentrated in the central and western parts of the study area. Total rainfall used was 436.7 mm, and the maximum runoff coefficient at pixel scale was 0.59. From 1984 to 2015, average direct runoff depths and total direct runoff volume presented an overall increased trend, from 54.79 mm to 62.40 mm, and 63.62 × 10^6^ to 72.31 × 10^6^ m^3^, respectively (Table 3 and Table 4). Average direct runoff depths gradually decreased from Zone 1 to 4, and were respectively 148.41 mm, 113.66 mm, 78.50 mm and 45.51 mm in 2015. The direct runoff is influenced by various factors. The direct runoff volume changed dramatically from 1984 to 2015, with change varying by area. The most important influence on direct runoff change was urban surface change caused by urbanization. Urbanization can cause a large amount of pervious surface to turn into impervious surface, changing the hydrologic cycle and reducing infiltration rates [45,46,47].

Most of the four-ring area had low runoff risk (904.40 km^2^), about 76.15% of the entire study area. The extremely high runoff risk area was also large, 119.47 km^2^ (10.06% of the entire area). Zone 4 had the largest extremely high runoff risk area (54.35 km^2^), whereas Zone 1 had the largest proportion of extremely high runoff risk area (27.58% of the zone).

Compared with runoff risk in 1984, the low runoff risk area in 2015 was reduced by a total of 150.92 km^2^, with the exception being Zone 1 with an increase of 3.80 km^2^ (Table 5). Medium and high runoff risk areas generally increased. The extremely high runoff risk areas of Zones 1 and 2 declined by 3.69 and 6.52 km^2^, but Zones 3 and 4 increased. As a result, the four-ring area had a 44.18 km^2^ increase in extremely high runoff risk area from 1984 to 2015.

### 3.3. Variation Trends of Direct Runoff at Regional Scale

**Figure 3:**
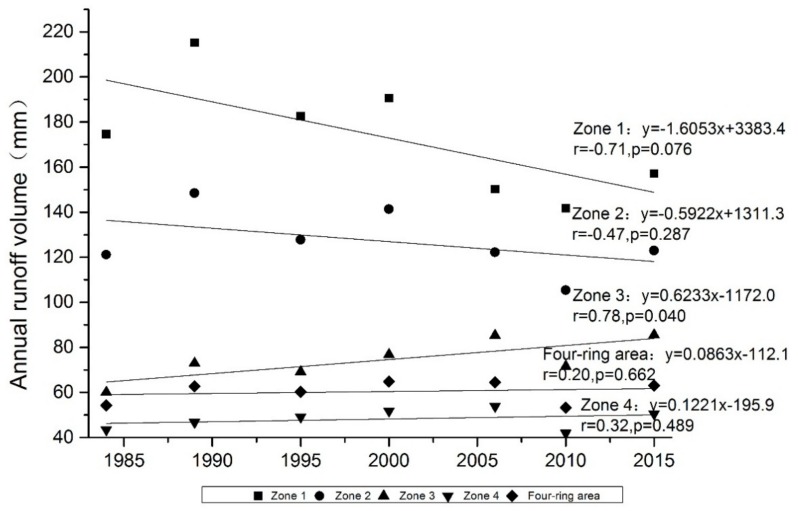
Trends of direct runoff at regional scale.

Analysis of this regression of direct rainfall runoffs for the entire study area shows that direct runoff of the four-ring area had weak growth (*b* > 0) at a rate of 0.086 mm year^−1^ (*p* = 0.662) from 1984 to 2015 (Figure 3). Urban sprawl of Shenyang gradually expanded from Zones 1 to 4, and the trends of direct runoff varied between the urban inner area to suburban area. Zone 1 is the core of the urbanized area, and direct runoff there decreased by 1.61 mm year^−1^ (*p* = 0.076). Direct runoff of Zone 2 maintained a decreasing trend, but the rate of decrease was lower compared with that of Zone 1, at 0.59 mm year^−1^ (*p* = 0.287). Zone 3 is an active area of urban development, where there are a large number of new residential communities and industrial buildings. That zone had a significant increasing trend at a rate of 0.62 mm year^−1^ (*p* = 0.662). Zone 4 is a suburban area where the city has expanded in recent years, where there is much agricultural land, forest land and undeveloped open space. The direct runoff volume of that zone increased by 0.12 mm year^−1^ (*p* = 0.489). The average direct runoff depth decline from Zones 1 to 4 mainly resulted from the impervious surface ratio decrease from the inner urban area to suburban areas. From 1984 to 2015, the direct runoff of Zones 1 and 2 gradually decreased. This may be mainly attributed to the increase of green area in the inner urban area, which can greatly increase the area of pervious surface. With the development of the social economy, more and more people pay attention to green space, so new residential communities tend to have more vegetation. From 2002 to 2010, the Shenyang government had implemented the Tiexi District transformation plan. A large number of factories were moved to suburban area from inner urban area, and many places have become residential, commercial or government areas with a large proportion of green areas. Since 2010, the urban renewal projects have been implemented, and many old districts in Zones 1 and 2 have been renovated.

### 3.4. Trends of Direct Runoff at Pixel Scale

#### 3.4.1. Trend and Extent

**Figure 4:**
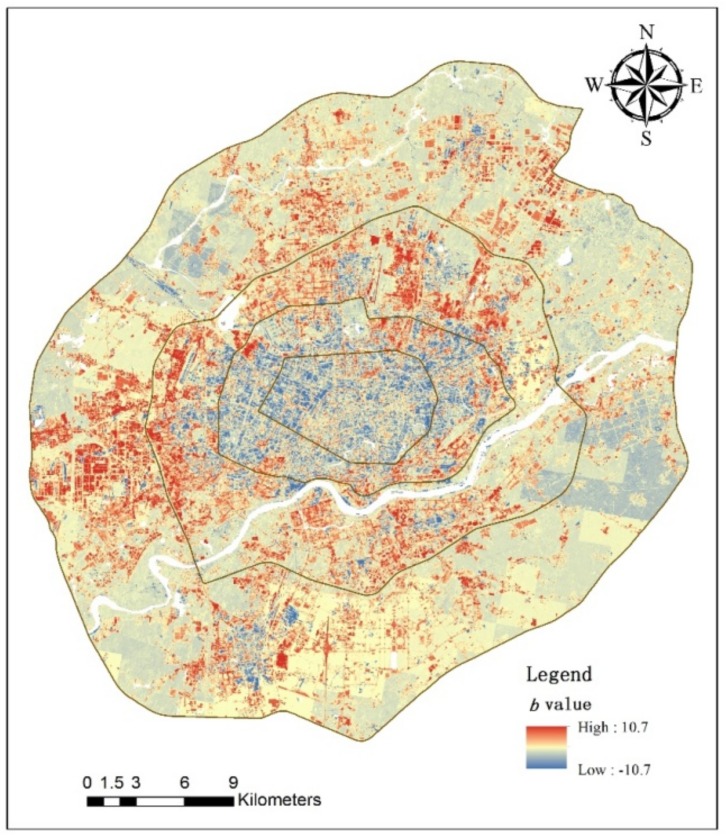
*b*-Value of direct runoff at pixel scale.

Spatial trends of direct runoff at pixel scale were analyzed by ordinary least-squares regression. Trends of direct runoff show strong spatial heterogeneity based on per-pixel analysis (Figure 4). Direct runoff in about 62.5% of the study (four-ring) area gradually decreased from 1984 to 2015 (*b* < 0), while only 37.5% of the area had a gradual increase (*b* > 0). Pixels with increasing trends of direct runoff from 1984 to 2015 were mainly in the west, north and south of Zones 3 and 4. With urban expansion, urban land use changed, and large amounts of farmland and non-construction land were converted to urban land. This increased the area of impervious surfaces, thereby increasing direct runoff. The north and south of Zones 3 and 4 are the Shenbei and Hunnan new development areas. As two important new development areas of Shenyang, they have had rapid urban expansion in the last 20 years. The west of Zones 3 and 4 are new industrial areas, where many industrial enterprises have moved from the urban core (Zones 1 and 2) since the implementation of industrial zone reform policy. The decreasing trend area was mainly in Zones 1 and 2, the urban core. This is mainly because of the Tiexi District transformation plan and urban renewal projects, which have increased the green area of residential districts and greenbelts of urban roads in urban core area.

**Figure 5:**
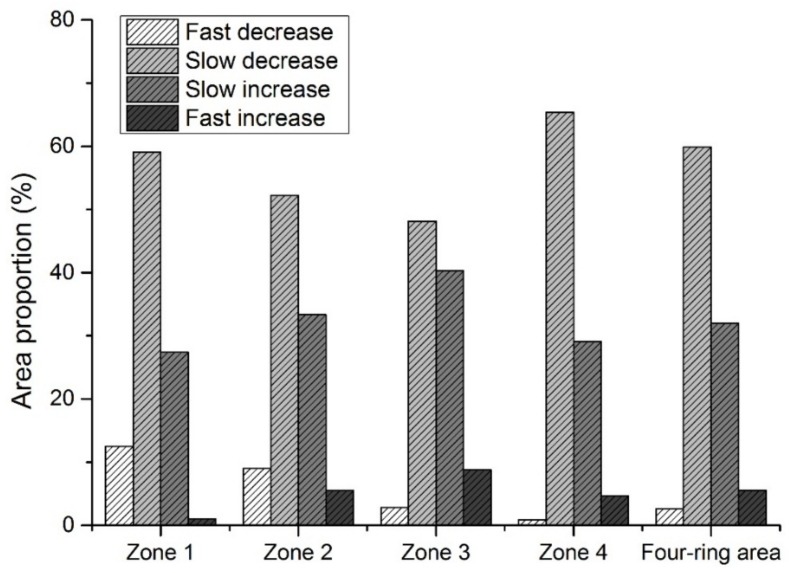
Extent of direct runoff trend in different zones.

The area of slow decrease level had the largest area proportion in either the entire study area or the four zones (Figure 5). In the four-ring area, direct runoff in about 60% of all pixels had a slow decrease from 1984 to 2015, and the fast decrease and fast increase areas accounted for 2.6% and 5.5% of the entire area. Area percentages of fast decrease gradually declined from Zones 1 to 4, with 12.5%, 9.0%, 2.8% and 0.9%, respectively, with corresponding areas 7.0 km^2^, 9.2 km^2^, 7.9 km^2^ and 6.6 km^2^. Zone 1 had the largest percentage of fast decrease, and Zone 2 the largest area of fast decrease. The largest area percentages of fast increase were in Zone 3 (8.7%), followed by Zone 2 (5.5%), Zone 4 (4.7%) and Zone 1 (1.1%), Corresponding areas were 24.3 km^2^, 5.6 km^2^, 35.2 km^2^ and 0.6 km^2^, respectively. Zone 3 had both the largest percentage and area of fastest increase.

#### 3.4.2. Significance Level Analysis

**Figure 6:**
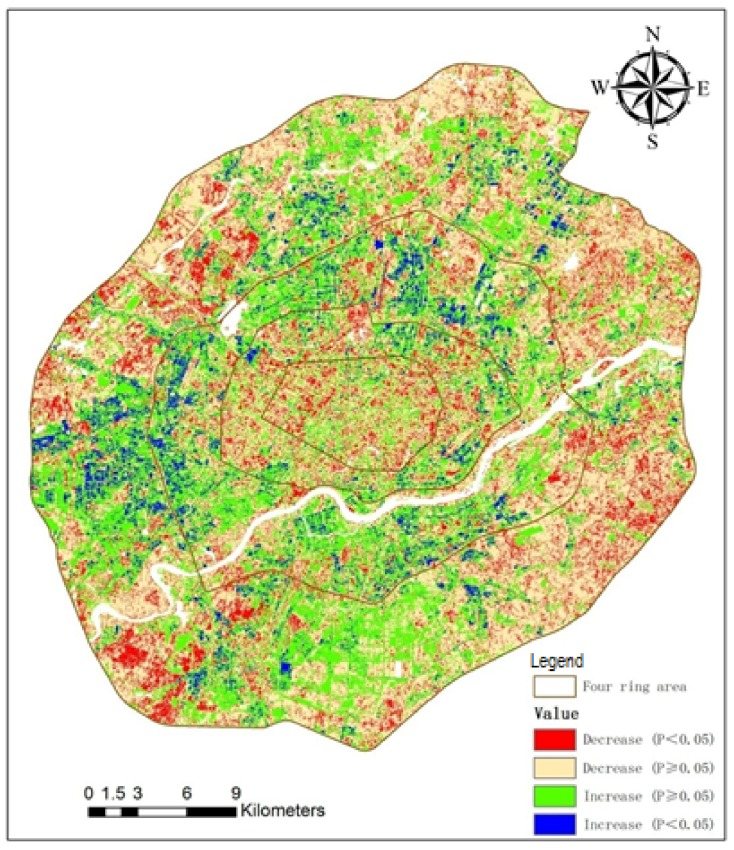
Classification of direct runoff trends at five percent significance level.

From Figure 6 and Table 6, we see that direct runoff trends are not significant (*p* ≥ 0.05) for most areas. Direct runoff trends are at the five percent significance level in only 20–25% of the area. In the entire four-ring area, the significant decrease area (*p* < 0.05) was larger than the significant increase area (*p* < 0.05), with area ratios 14.66% and 6.30%, respectively. The urban core (Zone 1) had the largest ratio of significant decrease area (20.69%) and the smallest ratio of significant increase (2.44%), which means that the direct runoff volume gradually declined in the urban core. Zone 3 had the largest ratio of significant increase area (10%) and the smallest ratio of significant decrease (10.96%), which means that this area had the most active urban development and amount of pervious land converted to impervious building surfaces.

## 4. Conclusions

In this study, we presented a very effective and efficient method to evaluate the direct runoff volume of the four-ring area in Shenyang. Though analyzing trends of direct runoff from 1984 to 2015 at different scales, we ascertained that total direct runoff volume of the study area increased from 6.36 × 10^7^ to 7.23 × 10^7^ m^3^ from 1984 to 2015. Average direct runoff depths gradually decreased from the inner core to suburban area. The low runoff risk area in 2015 was generally reduced compared with 1984. Extremely high runoff risk areas of Zones 1 and 2 decreased, while Zones 3 and 4 increased. Zones 1 and 2 had tendencies of decreasing direct runoff volume and risks, while those of Zones 3 and 4 gradually increased at both regional and pixel scales. In the four-ring area, pixels with increasing trends of direct runoff from 1984 to 2015 were mainly in the west, north and south of Zones 3 and 4. Direct runoff in about 60% of all pixels had a weak decreasing trend from 1984 to 2015, and those with fast decrease and fast area increase accounted for 2.6% and 5.5% of the study area. The area of significant decrease (*p* < 0.05) was larger than that of significant increase (*p* < 0.05), with area ratios 14.66% and 6.30%, respectively.

Urban surfaces are very complex and runoff coefficients for different surface types are very different. Thus, simplifying the urban surface as three basic types make calculation easy, but this increases uncertainty in the predicted direct runoff. In this study, CN values of different kinds of land use types were assigned according to the lookup table of TR-55, which may be very different from the actual CN value. Therefore, the estimated runoff will be more accurate if the use of local CN values or measured data validation is adopted.

Using this method, we could confirm hotspots of direct runoff in the city, which can provide a powerful basis for location selection and adjustment of green infrastructure. By using satellite images from different years, trends of direct runoff and flood-prone areas caused by the construction of industrial and residential areas may be recognized. Our method is an efficient tool to guide city management by the government and planning departments.

## Figures and Tables

**Table 1:** Vegetation classification and values of vegetation curve number (*CN_V_*) in dry antecedent moisture condition (AMC-I).

Vegetation Type		NDVI Range	*CN_V_*			
			A	B	C	D
Vegetated	Good Condition	NDVI ≥ 0.7	21	41	55	63
	Fair Condition	0.4 ≤ NDVI < 0.7	30	50	62	68
	Poor Condition	0 ≤ NDVI < 0.4	48	62	72	76
Non-Vegetated		NDVI < 0	59	72	80	85

NDVI: Normalized Difference Vegetation Index.

**Table 2:** Soil texture classification and values of soil curve number (*CN_S_*) in AMC-I.

Soil Type	Soil Texture	*CN_S_*
A	Sand ≥ 50% and clay ≤ 10%	59
B	Sand ≥ 50% and clay > 10%	72
C	Sand < 50% and clay ≤ 40%	80
D	Sand < 50% and clay > 40%	85

**Table 3:** Runoff depth in different regions from 1984 to 2015 (mm).

Zones	1984	1989	1995	2000	2006	2010	2015
Zone 1	165.32	208.79	175.7	182.19	137.89	134.20	148.41
Zone 2	109.86	139.17	119.17	131.94	110.18	97.40	113.66
Zone 3	55.12	68.35	64.01	71.13	76.64	66.30	78.50
Zone 4	41.55	45.23	47.07	49.85	49.65	39.51	45.51
Four-ring area	54.79	64.21	61.57	66.27	64.01	53.86	62.40

**Table 4:** Runoff volume in different regions from 1984 to 2015 (10^6^ m^3^).

Zones	1984	1989	1995	2000	2006	2010	2015
Zone 1	6.97	8.80	7.41	7.68	5.81	5.66	6.26
Zone 2	9.51	12.05	10.32	11.43	9.54	8.44	9.84
Zone 3	13.95	17.30	16.20	18.00	19.40	16.78	19.87
Zone 4	33.18	36.12	37.59	39.81	39.65	31.55	36.34
Four-ring area	63.62	74.28	71.52	76.92	74.41	62.43	72.31

**Table 5:** Variation of regional runoff risk from 1984 to 2015 (km^2^).

Zones	Low Runoff Risk		Medium Runoff Risk		High Runoff Risk		Extremely High Runoff Risk	
	Area in 2015	Area Change (2015–1984)	Area in 2015	Area Change (2015–1984)	Area in 2015	Area Change (2015–1984)	Area in 2015	Area Change (2015–1984)
Zone 1	19.68	3.80	10.46	−0.14	11.11	0.03	14.93	−3.69
Zone 2	55.31	−7.43	16.44	7.82	13.85	6.12	17.37	−6.52
Zone 3	197.20	−53.51	27.42	21.63	20.78	16.52	32.82	15.37
Zone 4	632.22	−93.78	38.04	32.51	25.63	22.28	54.35	39.00
Four−ring area	904.40	−150.92	92.36	61.80	71.37	44.95	119.47	44.18

**Table 6:** Area ratio of direct runoff trend by area.

Area	Decrease Ratio (*p* < 0.05)	Decrease Ratio (*p* ≥ 0.05)	Increase Ratio (*p* ≥ 0.05)	Increase Ratio (*p* < 0.05)
Zone 1	20.69	50.89	25.98	2.44
Zone 2	16.81	44.39	32.09	6.70
Zone 3	10.96	40.00	39.04	10.00
Zone 4	15.28	50.95	28.60	5.16
Four-ring area	14.66	47.82	31.22	6.30
